# 

**Published:** 1896-04-25

**Authors:** 


					? THE {HOSPITAL. 'ABSOLUTELY PURE, therefore BEST, April 25th, 1896.
CADBURY'S w v w CADBURY'S
cocoa rf INf |p cocoa
\j
'Skbf<^i^,/!ihsst p?'"y " ^ NO alkalies used.'
? ? rlCttJ>;c
?
No. 500.-* Vol. XX.
Saturday,
April 25th, 1896.
WITH EXTRA NURSING SUPPLEMENT. ?
[Registered at the General Post Ofltas aa a Newspaper for Monthly Parts, 6d.; post free, 8d
transmission in the United Kingdom and Abroaa.] Ditto per Annum, 8s.6d., post free.

				

